# Performance of the 2021 Estimated Glomerular Filtration Rate CKD-EPI Refit and the European Kidney Function Consortium (EKFC) Formulas

**DOI:** 10.3390/diagnostics15081047

**Published:** 2025-04-21

**Authors:** Evelyn O. Ilori, Casey R. Cai, Fatou Sahor, Brianna Wilson, Tanooha Veeramachaneni, Samir M. Parikh, Ibrahim A. Hashim

**Affiliations:** 1 Department of Pathology, University of Texas Southwestern Medical Center, Dallas, TX 75390, USA; 2 Department of Pathology, Parkland Health, Dallas, TX 75235, USA; 3 Department of Psychiatry, Vanderbilt University Medical Center, Nashville, TN 37232, USA; 4 Department of Internal Medicine, University of Texas Southwestern Medical Center, Dallas, TX 75390, USA

**Keywords:** eGFR, race, CKD-EPI, REFIT, EKFC, chronic kidney disease

## Abstract

**Background**: The glomerular filtration rate (GFR) is a universal clinical measure central to assessing kidney function and to the management of kidney disorders. Several formulas for the estimation of GFR are in use. The European Kidney Function Consortium (EKFC) formula has been reported to more accurately estimate the GFR as compared to the Chronic Kidney Disease Epidemiology Collaboration (CKD-EPI) formula and its recent version (REFIT equation) in European and African populations. However, validation of the EKFC equation in diverse U.S. populations, especially the Black subpopulation, is needed. **Methods**: Data from the electronic medical records of 75,442 individuals presenting to a large safety net county hospital with measurements of serum creatinine and/or iohexol clearance studies were used to calculate the estimated GFR (eGFR) and to determine CKD stage using the various reported eGFR formulas. The correlation between eGFR and measured GFR was determined for each equation. **Results**: The median eGFR for Black participants using the CKD-EPI, REFIT, and EKFC formulas was 130.6 mL/min/1.73 m^2^, 82.0 mL/min/1.73 m^2^, and 80.6 mL/min/1.73 m^2^ (*p* < 0.001), respectively. For White participants, the median eGFR using the CKD-EPI, REFIT, and EKFC formulas was 145.3 mL/min/1.73 m^2^, 105.6 mL/min/1.73 m^2^, and 99.2 mL/min/1.73 m^2^, respectively (*p* < 0.001). The REFIT equation underestimates the mGFR in Black individuals at eGFR < 80 mL/min per 1.73 m^2^ and in White individuals at eGFR > 20 mL/min per 1.73 m^2^. In comparison, the EKFC equation underestimates the mGFR at eGFR > 20 mL/min per 1.73 m^2^ in both Black and White individuals. The REFIT equation had the least absolute median bias as compared to EKFC and CKD-EPI in both Black and White participants (*p* < 0.0001). The P30 of the REFIT and EKFC equations was not statistically different for either Black or White participants (*p* = 0.16, *p* = 0.37). **Conclusions**: Although the accuracies (P30) of the EKFC and REFIT equations are not statistically significant (*p* = 0.16 and 0.37, Black and White individuals, respectively), adopting the EKFC formula in Americans requires the evaluation of each subpopulation. Both the EKFC and REFIT formulas underestimate the mGFR at a lower eGFR, which may have a direct impact on CKD classification for Black and White patients, with potentially significant implications for clinical management.

## 1. Introduction

Chronic kidney disease (CKD) affects over 800 million individuals worldwide, posing a major public health burden due to its significant contribution to morbidity and mortality [1]. Comorbidities such as hypertension, cardiovascular disease, and diabetes can further impair renal function, leading to a decline in the glomerular filtration rate (GFR) [2]. The GFR is the primary clinical measure of kidney function and is essential for diagnosing CKD, staging its severity, and guiding clinical decision-making and management [3]. While measured GFR (mGFR) using exogenous markers such as iothalamate or iohexol clearance remains the gold standard, its routine use is limited by cost, complexity, and availability [4]. Similarly, endogenous creatinine clearance, which relies on timed urine collections (often for 24 h), is inconvenient and prone to inaccuracies due to incomplete urine collection, variability in muscle mass, protein metabolism, technical interference in creatinine measurements, and renal tubular handling of creatinine [5]. Consequently, estimated GFR (eGFR) equations based on serum creatinine (Cr) remain easier to use and indispensable for the clinical assessment of kidney function.

The Chronic Kidney Disease Epidemiology Collaboration (CKD-EPI) equation, developed in the 2000s, has become the most widely used eGFR formula due to its improved accuracy compared to the earlier Modification of Diet in Renal Disease (MDRD) formula [6]. However, the CKD-EPI has limitations, particularly its adoption of patient race as a variable, which is widely recognized as a social construct rather than a biologic determinant of kidney function [7,8]. Furthermore, racial disparities in the early recognition of kidney disease and outcomes have been well documented, with Black and Hispanic populations presenting late and with more severe glomerular disease compared to White populations [9]. The CKD-EPI Refit (REFIT) equation was developed in 2021 to reduce bias by eliminating the race coefficient [8].

In parallel, the European Kidney Function Consortium developed an alternative formula (EKFC) that incorporates age and sex and utilizes a population-dependent normalization coefficient (Q) for improved precision [10]. The EKFC equation has been validated across a broad adult age range among Europeans and Africans, including a few studies among Asians [10,11,12,13,14]. Although it is widely used, however, there have been few studies examining the impact of the EKFC equation in Americans, especially the Black American population that is at significant risk of advanced CKD [15,16]. This study compares the performance of commonly used eGFR equations with regards to accuracy and clinical impact on patients at a large, tertiary academic medical center also serving as a safety net county hospital.

## 2. Materials & Methods

This observational study was approved by the University of Texas Southwestern Institutional Review Board and the Parkland Hospital Research Office (IRB STU-2021-1030).

### 2.1. Patient Population

Clinical laboratory results and demographics (self-declared race and gender) data were obtained from the electronic health record system (Epic^®^, Epic Systems, Verona, WI, USA) of patients ≥ 18 years old presenting to a large safety net county hospital with clinically ordered serum creatinine and/or GFR estimation via iohexol measurement from August 2019 to November 2020.

Of the 78,472 patients eligible, 75,442 Black and White individuals were included in this study. Other racial groups aside from White and Black were excluded from this study due to relatively small numbers. Female patients represented 66.8% of Black individuals and 70.3% of the White study population.

### 2.2. Estimated GFR Determination (eGFR)

Serum creatinine (Cr) was measured enzymatically (via creatinine kinase and sarcosine oxidase) using COBAS^®^ automated instrument (Roche Diagnostics, Indianapolis, IN, USA). The assay is standardized and traceable to the isotope-dilution mass spectrometry. eGFR was calculated using the following equations.

CKD-EPI:

eGFR = 141 × min(Cr/k, 1)^a^ × max(Cr/k, 1)^−1.209^ × 0.993 × 1.018 (if female) × 1.159 (if Black)

where (k) was 0.7 for women and 0.9 for men, (a) being −0.329 for women and −0.411 for men [3].

REFIT:

eGFR = 142 × min(Cr/A, 1)^B^ × max(Cr/A, 1)^−1.2^ × 0.9938^Age^ × (1.012 if female)

where (A) was 0.7 for women and 0.9 for men, and (B) was −0.241 for women and −0.302 for men [16].

EKFC [17]:

eGFR = 107.3 × (Cr/Q)^−0.322^ for adult male and female ≤ 40 years old and (Cr/Q) < 1.0

eGFR = 107.3 × (Cr/Q)^−1.132^ for adult male and female ≤ 40 years old and (Cr/Q) ≥ 1.0

eGFR = 107.3 × (Cr/Q)^−0.322^ × 0.990^(Age−40)^ for adult male and female > 40 years old and (Cr/Q) < 1.0

eGFR = 107.3 × (Cr/Q)^−1.132^ × 0.990^(Age−40)^ for adult male and female > 40 years old and (Cr/Q) ≥ 1.0

(Q) values were 1.0 mg/dL (Black male), 0.93 mg/dL (White male), and 0.73 mg/dL (Black and White female). Creatinine was expressed as mg/dL.

### 2.3. Measured GFR (mGFR)

GFR was measured in 171 of the study participants as part of their routine medical care (e.g., assessment as candidate donors for renal transplant). Iohexol was administered intravenously and its clearance determined as previously described using an in-house liquid chromatography–mass spectroscopy method. The assay’s analytical measurement ranged from 5 to 1000 mg/mL with imprecision < 10%. The two-compartment model was used to calculate the GFR [18].

### 2.4. Kidney Disease Staging

CKD stages were assigned using the Kidney Disease Improving Global Outcomes (KDIGO) guidelines (2012) [19]. The CKD stage was determined using the eGFR calculated using the CKD-EPI, REFIT, and EKFC equations and compared.

### 2.5. Statistical Analyses

The 2-sided Pearson’s Chi square analysis was used to assess the significance of CKD reclassification based on the respective eGFR equations. The mean comparative difference (bias) is calculated by subtracting the eGFR from the mGFR, along with the 95% confidence interval (95%CI). The P30 accuracy was also calculated with 95%CI. The KDIGO reports P30 > 75% as adequate for an eGFR equation to be considered good for clinical decision [15]. All other *p* values were calculated using the paired *t* test. All statistical analyses were performed using the Version 25.0.2 NCSS 2025 Statistical Software (NCSS, LLC. Kaysville, UT, USA, https://www.ncss.com/software/ncss/, accessed on 15 April 2025).

## 3. Results

The demographic and laboratory data for 75,442 individuals, comprising 25,003 Black individuals and 50,439 White individuals, included in the study are summarized in Table 1. The median ages for Black (54 years) and White (49 years) patients are statistically different (*p* < 0.0001). Females represented 66.8% of the Black and 70.3% of the White study participants. The median serum creatinine level is significantly higher in Black participants, with the eGFR values calculated using the CKD-EPI, REFIT, and EKFC equations being lower, as expected, in the Black population (*p* < 0.0001) (Table 1). The EKFC formula resulted in the lowest eGFR values for both Black and White individuals, followed by the REFIT formula.

### 3.1. Reclassification of CKD Stages (As Defined by KDIGO) [15]

The clinical impact of applying the different eGFR equations was evaluated by comparing the obtained CKD stage with the respective eGFR formula (Table 2A). Among Black participants, the REFIT equation reclassified about 80.3% of patients to a higher CKD stage compared to when using the CKD-EPI equation (Table 2A). However, applying the EKFC equation resulted in the reclassification of only 6.5% of Black patients to a higher stage and 8.3% to a lower stage when compared to the REFIT equation (Table 2B).

However, among White participants, using the REFIT equation reclassified 61.0% on average to a higher CKD stage compared to using CKD-EPI (Table 3A), whereas, when applying the EKFC formula, about 7.5% were reclassified to a higher CKD stage and only about 2.6% reclassified to a lower stage (Table 3B). The transitions were all statistically significant (*p* < 0.005).

### 3.2. Accuracy of eGFR as Compared to Measured GFR

**Figure 1:**
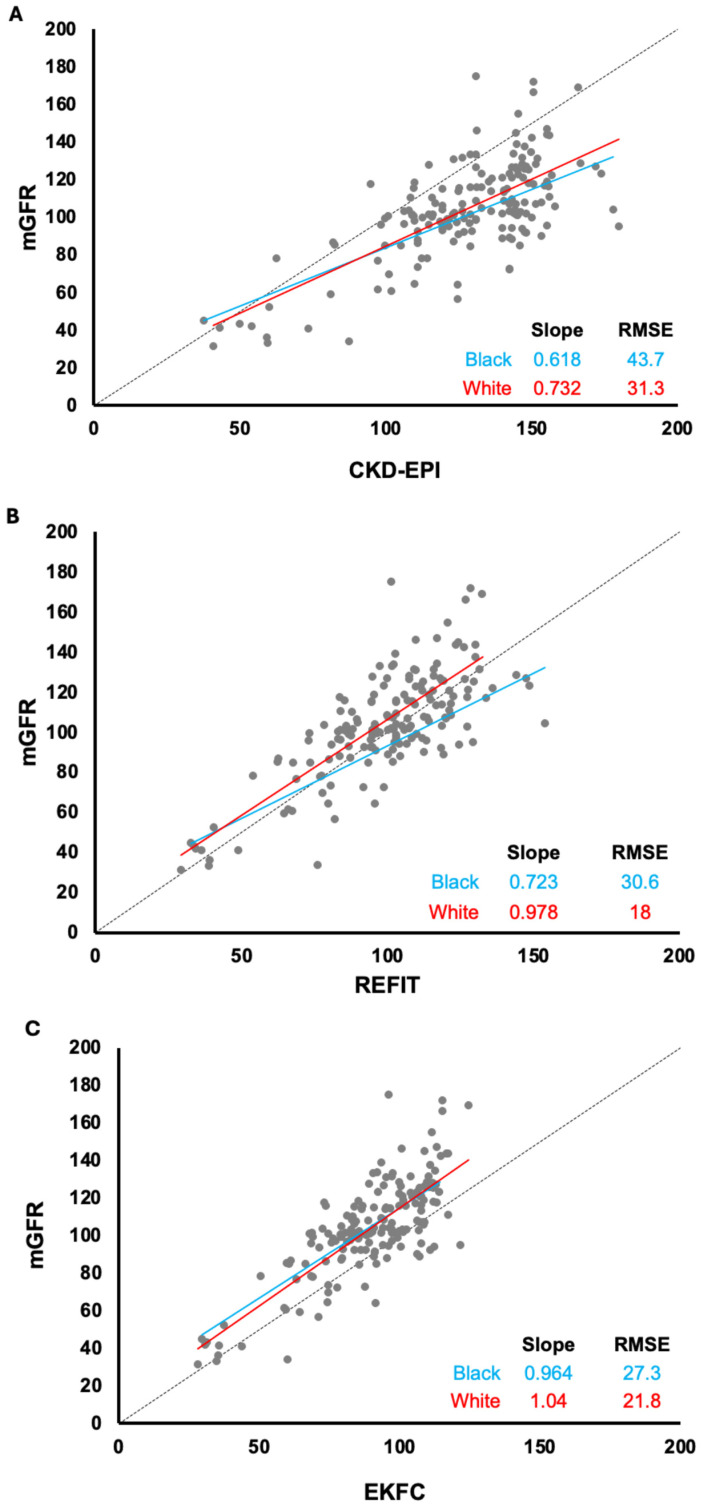
Correlation between population-specific (Black and White) measured GFR (mGFR) and corresponding estimated GFR (eGFR) when using (**A**) CKD-EPI, (**B**) REFIT, and (**C**) EKFC formulas. CKD-EPI: Chronic Kidney Disease Epidemiology Collaboration. REFIT: revised CKD-EPI formula without correction for race; EKFC: European Kidney Function Consortium. GFR is in mL/min/1.73 m^2^, (----) represents line of identity.

Measured GFR (mGFR) by iohexol was compared with estimated (eGFR) using the CKD-EPI, REFIT, and EKFC formulas for Black and White participants (Figure 1).

Among Black individuals, all three eGFR formulas underestimated GFR when compared to mGFR at eGFR < 60 mL/min per 1.73 m^2^. However, the CKD-EPI formula overestimated the GFR compared to the measured GFR (mGFR) among Black participants for eGFR > 60 mL/min per 1.73 m^2^ and among White participants for eGFR > 40 mL/min per 1.73 m^2^ (Figure 1A).

The REFIT equation underestimated the GFR compared to the mGFR among Black participants when eGFR < 80 mL/min per 1.73 m^2^ and overestimated the GFR compared to the mGFR when eGFR > 80 mL/min per 1.73 m^2^ (Figure 1B). Among the White population, the REFIT equation underestimated the GFR compared to the mGFR, irrespective of the eGFR values. Furthermore, the EKFC formula underestimated the GFR compared to the mGFR among both White and Black populations when eGFR > 20 mL/min per 1.73 m^2^ (Figure 1C).

Among the Black population, the line slope is similar for the CKD-EPI and REFIT equations, while the line slope is similar for the REFIT and EKFC equations among the White population.

The median bias between mGFR and eGFR was determined for all three equations in both racial populations (Table 4). Median GFR bias was significantly different between mGFR and eGFR as obtained by the different formulas—CKD-EPI and REFIT, and EKFC and REFIT—for both the Black and White groups (*p* < 0.0001 for all comparisons). Although the absolute median bias is >5 for all three eGFR equations for both subgroups, the REFIT equation results in the least absolute median bias.

When compared to CKD-EPI, the REFIT equation had a significantly higher P30 among both the Black and White populations (*p* = 0.0055 and *p* < 0.0001, respectively). However, although higher than that for the EKFC, the P30 for the REFIT equation is not statistically significant in either the Black (*p* = 0.16) or White (*p* = 0.37) subgroups.

## 4. Discussion

More than one in seven adults in the United States is affected by chronic kidney disease, which makes accurate estimation of the glomerular filtration rate critical for diagnosis and management, particularly in populations where disparities in CKD prevalence and outcomes persist [20]. This study evaluated the performance of three widely used creatinine-based eGFR equations—CKD-EPI, REFIT, and EKFC—in a large cohort of Black and White individuals. Notably, our institution is a safety net county hospital that serves a unique population of patients with a high burden of chronic diseases and morbidity. Our findings provide important insights into the strengths and limitations of these equations and contribute to the ongoing debate on the best approach to GFR estimation.

### 4.1. Performance of the EKFC Equation in Our Cohort

The EKFC equation was developed to improve accuracy across diverse populations by incorporating age, sex, and serum creatinine that is normalized using a Q value [10]. While earlier studies have reported the improved accuracy of the EKFC equation in Black and non-Black populations in Europe, Africa, and Asia, limited validation studies have been conducted in the United States, particularly among U.S.-based Black populations [15].

In our cohort, the EKFC equation consistently underestimated the mGFR in both Black and White individuals. Although the REFIT equation yielded the smallest median bias in both racial subgroups, it also underestimated the mGFR in Black participants at eGFR < 80 mL/min per 1.73 m^2^. Underestimation of the glomerular filtration rate could result in a significant increase in nephrology referrals, overtreatment, patient harm, and medical waste. A recent evaluation of the EKFC equation in over 12,000 adults from multiple U.S. cohorts reported less median bias for both the race-free and population-specific versions of the EKFC equation as compared to the REFIT, with similar P30 across equations [15]. Our study shows that institutions in the United States with unique patient populations should evaluate the effectiveness of the EKFC equation before adoption.

The clinical impact of transitioning to the EKFC equation from the REFIT equation is highlighted by the reclassification of CKD stages for both Black and White participants. For example, a mean of 6.5% of Black participants were reclassified to a higher CKD stage and 8.3% reclassified to a lower stage. For White participants, 7.5% were reclassified to a higher CKD stage, and 2.6% were reclassified to a lower stage. These shifts in CKD staging may have significant clinical implications, as they can influence decisions regarding medication dosing, nephrology referral, and eligibility for kidney transplantation for individual patients. Our study highlights the importance of selecting an eGFR equation that aligns with the clinical context and is appropriate for the patient population being served.

### 4.2. Comparison of the REFIT and CKD-EPI Equations

Our study demonstrates that the REFIT equation, which does not include a race correction factor, results in significantly more accurate eGFR compared to the race-based CKD-EPI formula in both Black and White populations. Black individuals are disproportionately affected by CKD and experience worse outcomes compared to White individuals, including in pediatric populations [21]. Eliminating the race factor from the eGFR equation may facilitate earlier identification of renal impairment in Black patients, enabling timely intervention and potentially preventing further kidney dysfunction. The clinical impact of transitioning from the CKD-EPI to the REFIT equation is highlighted by the reclassification of an average of 80.3% and 61.0% of Black and White participants, respectively, to a higher CKD stage. The median bias and P30 are also significantly improved using the REFIT as compared to the CKD-EPI equation. An epidemiological study using the NHANES database and examining the potential effects of switching to the REFIT equation from the CKD-EPI equation showed an 11% increase in the number of chronic kidney disease diagnoses among Black adults and a 20% decrease among non-Black adults [6]. Interestingly, the trend affects kidney transplant eligibility at CKD stage 3b and higher, with a 9% increase among Black adults as compared to an 8% decrease among non-Black adults. This study underscores the value of removing race as a component of the eGFR calculation, promoting the transition from the CKD-EPI to the REFIT equation. This study also highlights the discrepancy in CKD classification among the populations studied. Adoption of the REFIT formula will aid in the earlier identification of patients with CKD and, thus, the institution of therapy. Future studies will examine outcomes with respect to the adoption of the revised formula.

### 4.3. Discourse on the Accuracy of Creatinine-Based Equations

This study aligns with prior studies suggesting that creatinine-based equations demonstrate imprecision, particularly due to factors that influence serum creatinine levels, and advocating the use of multiple filtration markers such as creatinine and cystatin C instead of solely creatinine in calculating eGFR to lessen the impact of muscle mass in eGFR calculations [16]. While cystatin-based equations have shown promise in improving accuracy and reducing racial bias, creatinine remains widely used due to its accessibility and cost-effectiveness. A recent study of >4000 adults indicated that both REFIT and EKFC are effective depending on the population being evaluated [22].

### 4.4. Strengths and Limitations

This study has several strengths, including its large sample size, especially the large population of Black individuals, served by our county hospital. Our findings provide valuable insights into the clinical impact of three widely used eGFR equations in a population with advanced and complex medical conditions. Notably, we showed that the EKFC equation underestimates the mGFR while the REFIT tends to underestimate the mGFR at eGFR < 80 mL/min per 1.73 m^2^ in the Black population. This could potentially result in the overdiagnosis of CKD in this subgroup. These findings underscore the importance of careful clinical interpretation of eGFR values. However, our study also has limitations. First, the retrospective design may introduce selection bias, as participants with available iohexol measurements may differ from the general population. Secondly, we did not evaluate cystatin C-based equations, which have shown promise in reducing racial bias in GFR estimation [15]. This study was conducted at a single institution, which also impacted the number of patients with urinary iohexol clearance data. Although iohexol is considered one of the gold standards for mGFR, studies have reported imprecision, which may result in increased bias [23].

## 5. Conclusions and Future Directions

In conclusion, our study provides novel insights into the performance of the EKFC, REFIT, and CKD-EPI equations in a patient population at a county hospital with a high proportion of Black patients. Both the EKFC and REFIT equations demonstrated superiority to the CKD-EPI equation, with the REFIT showing the least bias and highest accuracy in both Black and White participants. Screening and diagnostic tools based on inherent biology are needed to overcome the imprecision of race-based medicine and address health disparities in minority populations. Future studies should continue to explore eGFR equations that incorporate biomarkers aside from creatinine, such as cystatin C. Also, longitudinal studies are needed to assess the clinical impact of eGFR reclassification on patient outcomes, especially with the adoption of the EKFC equation in U.S. populations.

## Figures and Tables

**Table 1:** Study population demographics and eGFR obtained using the CKD-EPI, REFIT, and EKFC equations. Female patients represented 66.8% of the Black and 70.3% of the White study population. Serum creatinine is expressed as mg/dL, eGFR is expressed as mL/min per 1.73 m^2^. eGFR: estimated glomerular filtration rate; CKD-EPI: Chronic Kidney Disease Epidemiology Collaboration; REFIT: revised CKD-EPI formula without correction for race; EKFC: European Kidney Function Consortium. Age is in years. Values are expressed as median (range).

Race			
Median (Range)	Black	White	*p* Value
***N***	25,003	50,439	
**Age**	54 (19–103)	49 (19–100)	<0.0001
**Serum Creatinine**	0.93 (0.17–24.14)	0.70 (0.1–16.03)	<0.0001
**eGFR CKD-EPI**	130.6 (3.0–321.9)	145.3 (3.4–270.4)	<0.0001
**eGFR REFIT**	82.0 (1.9–168.8)	105.6 (2.7–194.2)	<0.0001
**eGFR EKFC**	80.6 (2.5–156.8)	99.2 (3.2–203.5)	<0.0001

**Table 2:** (**A**) Number of Black patients (percentage of patients) at each CKD stage as determined using the CKD-EPI and its REFIT versions. Patients transitioned to a higher CKD stage when using the REFIT formula. CKD-EPI: Chronic Kidney Disease Epidemiology Collaboration. REFIT: revised CKD-EPI formula without correction for race; EKFC: European Kidney Function Consortium. (**B**): Number of Black patients (percentage of patients) at each CKD stage as determined using the CKD-EPI and its REFIT versions. Few patients transitioned to a higher CKD stage when using the EKFC formula.

(**A**)							
REFIT							
CKD-EPI		Stage 1	Stage 2	Stage 3a	Stage 3b	Stage 4	Stage 5
	Stage 1	10,002	9576 (46.3%)	1113 (5.4%)	1 (<0.1%)		
	Stage 2		89	1654 (61.5%)	947 (35.2%)		
	Stage 3a			0	421 (69.2%)	187 (30.8%)	
	Stage 3b				13	400 (96.9%)	
	Stage 4					153	196 (56.2%)
	Stage 5						194
(**B**)							
EKFC							
REFIT		Stage 1	Stage 2	Stage 3a	Stage 3b	Stage 4	Stage 5
	Stage 1 (*n* = 10,002)	8530	1472 (14.7%)				
	Stage 2 (*n* = 9665)	417 (4.3%)	8697	551 (5.7%)			
	Stage 3a (*n* = 2767)		254 (9.2%)	2296	217 (7.8%)		
	Stage 3b (*n* = 1382)			101 (7.3%)	1226	55 (4.0%)	
	Stage 4 (*n* = 740)				50 (6.8%)	686	4 (0.5%)
	Stage 5 (*n* = 390)					49 (13.8%)	341

CKD-EPI: Chronic Kidney Disease Epidemiology Collaboration. REFIT: revised CKD-EPI formula without correction for race; EKFC: European Kidney Function Consortium.

**Table 3:** (**A**): Number of White patients (percentage of patients) at each CKD stage as determined using the CKD-EPI and its REFIT equations. Patients transitioned to a higher CKD stage when using the REFIT formula. CKD-EPI: Chronic Kidney Disease Epidemiology Collaboration. REFIT: revised CKD-EPI formula without correction for race; EKFC: European Kidney Function Consortium. (**B**): Number of White patients (percentage of patients) at each CKD stage as determined using the EKFC and its REFIT versions. Few patients transitioned to a higher CKD stage when using the EKFC formula.

(**A**)							
REFIT							
CKD-EPI		Stage 1	Stage 2	Stage 3a	Stage 3b	Stage 4	Stage 5
	Stage 1 (*n* = 44,904)	36,427	8368 (18.6%)	109 (0.2%)			
	Stage 2 (*n* = 3271)		861	1999 (61.1%)	411 (13.5%)		
	Stage 3a (*n* = 884)			31	834 (94.3%)	19 (2.1%)	
	Stage 3b (*n* = 535)				129	406 (75.9%)	
	Stage 4 (*n* = 473)					288	185 (39.1%)
	Stage 5 (*n* = 270)						270
(**B**)							
EKFC							
REFIT		Stage 1	Stage 2	Stage 3a	Stage 3b	Stage 4	Stage 5
	Stage 1 (*n* = 36,427)	32,219	4208 (11.6%)				
	Stage 2 (*n* = 9229)	30 (0.3%)	8477	722 (7.8%)			
	Stage 3a (*n* = 2139)		20 (0.9%)	1858	261 (12.2%)		
	Stage 3b (*n* = 1374)			24 (1.7%)	1280	70 (5.1%)	
	Stage 4 (*n* = 713)				27 (3.8%)	680	6 (0.8%)
	Stage 5 (*n* = 455)					29 (6.4%)	426

CKD-EPI: Chronic Kidney Disease Epidemiology Collaboration. REFIT: revised CKD-EPI formula without correction for race; EKFC: European Kidney Function Consortium.

**Table 4:** Median bias and accuracy (P30) among both Black and White populations when comparing eGFR estimated using either the CKD-EPI or the EKFC formulas with that obtained by the REFIT formula. *p* value a compares REFIT and CKD-EPI; *p* value b compares REFIT and EKFC.

Median Bias (95%CI)	CKD-EPI	REFIT	EKFC	*p* Value a	*p* Value b
Black	−27.7 (−36.3, −22.2)	−11.2 (−17.1, −5.5)	16.9 (10.1, 20.4)	<0.0001	<0.0001
White	−22.3 (−26.8, −20.0)	6.2 (3.8, 9.2)	11.5 (8.9, 14.2)	<0.0001	<0.0001
**P30 (95%CI)**					
Black	66.7 (58.9, 74.4)	91.7 (87.1, 96.2)	83.3 (77.2, 89.4)	0.0055	0.16
White	62.4 (54.6, 70.2)	93.3 (89.3, 97.3)	92.6 (88.4, 96.8)	<0.0001	0.37

## Data Availability

The data presented in this study are available on request from the corresponding author. The data are not publicly available because although study data were de-identified, combination of each individual data point may result in potential identification.
